# Immune checkpoint inhibitor‐induced enteritis assessed using capsule endoscopy

**DOI:** 10.1002/jgh3.12392

**Published:** 2020-07-20

**Authors:** Shinsuke Otagiri, Takehiko Katsurada, Kana Yamanashi, Kensuke Sakurai, Michiko T Sato, Jun Sakakibara‐Konishi, Naoya Sakamoto

**Affiliations:** ^1^ Department of Gastroenterology and Hepatology Hokkaido University Graduate School of Medicine Sapporo Japan; ^2^ First Department of Medicine Hokkaido University Hospital Sapporo Japan

**Keywords:** capsule endoscopy, immune‐related adverse event, intestinal disorder

## Abstract

We performed capsule endoscopy for a patient with immune checkpoint inhibitor‐induced enteritis and found multiple erosions or small ulcers in the small intestine. No reports demonstrated the effectiveness of capsule endoscopy for immune checkpoint inhibitor‐induced gastrointestinal adverse events, and our case suggests that capsule endoscopy may be useful to evaluate immune checkpoint inhibitor‐induced enteritis.

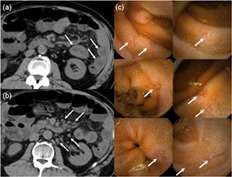

A 68‐year‐old man was admitted to our hospital due to liquid and black stools eight times a day, fever, and abdominal pain. He was diagnosed with malignant pleural mesothelioma (stage IV) and had been treated with nivolumab for 2 months before the admission. His serum C‐reactive protein (CRP) level increased to 17.1 mg/dL and creatinine level to 1.70 mg/dL. Computed tomography (CT) showed diffuse wall thickness of the jejunum (Fig. 1a) and lymph node swellings of the mesentery (Fig. 1b). Esophagogastroduodenoscopy (EGD) and colonoscopy (CS) showed several aphthas in the stomach and cecum. Results of fecal microbiological examination showed no apparent abnormalities, and fasting did not improve his symptoms or CRP levels; therefore, he was diagnosed with a severe immune checkpoint inhibitor (ICI)‐induced gastrointestinal (GI) adverse event (AE), and 60 mg/day prednisolone was initiated. His symptoms and increased CRP level immediately improved after the administration. As endoscopic findings were mild compared to his symptoms and CRP level, and CT scan images suggested small intestinal inflammation, capsule endoscopy was performed. Capsule endoscopy showed multiple erosions or small ulcers in the small intestine (Fig. 1c), these findings were more inflamed than the findings in the stomach or colon. He was discharged from our hospital 24 days after admission, and prednisolone dosage was decreased.

**Figure 1:**
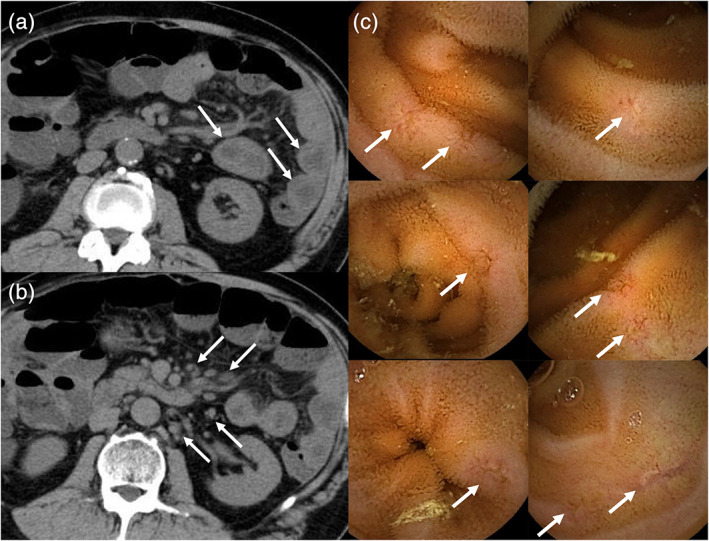
Computed tomography shows diffuse wall thickness of the jejunum (a, arrow) and lymph node swellings of the mesentery (b, arrow). Capsule endoscopy shows multiple erosions or small ulcers in the small intestine (c, arrow).

It is well known that ICIs have GI toxicity, and diarrhea occurs in 2.9–11.5% and colitis in 1.3–2.9% of patients treated with antiprogrammed cell death protein 1 antibody.^1^ EGD and CS are recommended to manage GI symptoms of patients treated with ICIs^1^, ^2^; however, no reports demonstrated the effectiveness of capsule endoscopy for ICI‐induced GI AEs.^3^ Our case suggests that capsule endoscopy may be used to evaluate GI AEs when EGD and CS show no conclusive findings, and multiple erosions or small ulcers may occur as ICI‐induced enteritis.
